# Sumoylation Regulates Nuclear Localization of Lipin-1α in Neuronal Cells

**DOI:** 10.1371/journal.pone.0007031

**Published:** 2009-09-15

**Authors:** Guang-Hui Liu, Larry Gerace

**Affiliations:** Department of Cell Biology, The Scripps Research Institute, La Jolla, California, United States of America; Texas Tech University Health Sciences Center, United States of America

## Abstract

Lipin-1 is a protein that has dual functions as a phosphatidic acid phosphohydrolase (PAP) and a nuclear transcriptional coactivator. It remains unknown how the nuclear localization and coactivator functions of lipin-1 are regulated. Here, we show that lipin-1 (including both the alpha and beta isoforms) is modified by sumoylation at two consensus sumoylation sites. We are unable to detect sumoylation of the related proteins lipin-2 and lipin-3. Lipin-1 is sumoylated at relatively high levels in brain, where lipin-1α is the predominant form. In cultured embryonic cortical neurons and SH-SY5Y neuronal cells, ectopically expressed lipin-1α is localized in both the nucleus and the cytoplasm, and the nuclear localization is abrogated by mutating the consensus sumyolation motifs. The sumoylation site mutant of lipin-1α loses the capacity to coactivate the transcriptional (co-) activators PGC-1α and MEF2, consistent with its nuclear exclusion. Thus, these results show that sumoylation facilitates the nuclear localization and transcriptional coactivator behavior of lipin-1α that we observe in cultured neuronal cells, and suggest that lipin-1α may act as a sumoylation-regulated transcriptional coactivator in brain.

## Introduction

Covalent attachment of the small ubiquitin-like modifiers (SUMOs) to lysine residues in target proteins, or sumoylation, is an important regulator of protein functions [1]–[4]. Sumoylation of target proteins is a multi-step process involving the E1-activating complex Aos1/Uba2 and the E2-conjugating enzyme Ubc9 in mammalian cells. These two suffice to drive SUMO conjugation *in vitro*. *In vivo*, sumoylation typically involves E3 factors, which enhance the specificity and rate of modification. SUMO modification usually is reversible, and multiple SUMO-specific proteases that remove the modification have been identified [5], [6]. The substrate lysines for sumoylation are typically found within the consensus sequence ΦKXE (Φ represents hydrophobic amino acids; K, lysine; X, any amino acid; E, glutamate). Cells express three major SUMO paralogs, SUMO-1, SUMO-2, and SUMO-3, with SUMO-2 and 3 being much more similar to each other than to SUMO-1 [2]. Many nuclear proteins are sumoylated and this has been linked to nuclear localization, transcriptional activation or repression, and other processes [7]-[9]. Proteins whose nuclear localization is facilitated by sumoylation include CtBP1 [10], CREB [11], Mdm2 [12] and KLF5 [13].

The protein encoded by the LPIN1 gene (lipin-1) has been implicated in the regulation of cellular lipid metabolism in a variety of tissues including adipose tissue [14], liver [15], muscle [16], and myelinated motor neurons [17]. LPIN1, was discovered using a positional cloning approach to identify the genetic mutation responsible for the fatty liver dystrophic (*fld*) mouse phenotype [18]. *Fld* mice exhibit life-long deficiency in adipocyte differentiation, peripheral neuropathy, circulating hyperlipidemia, and neonatal hepatic steatosis [19]–[21]. Conversely, transgenic lipin-1 overexpression in skeletal muscle or white adipose tissue of mouse exacerbates high-fat diet-induced obesity due to effects on adipocytes [16]. In higher eukaryotes, genes encoding three lipin family members (lipin-1, lipin-2, and lipin-3) have been identified [18]. In addition, two lipin-1 protein isoforms (lipin-1α and lipin-1β) are generated by alternative mRNA splicing [14], [22]. In humans, mutations in LPIN1 are associated with metabolic syndrome and type-2 diabetes [19], [23]. Both mammalian lipin-1 and its budding yeast homolog (*PAH1* or *SMP2*) were revealed to have enzymatic activity as a phosphatidic acid phosphohydrolase (PAP) [24]. PAP proteins catalyze the formation of diacylglycerol (DAG) from phosphatidic acid (PA) in the penultimate step in triglyceride (TAG) synthesis. Recently, several lines of evidences indicate that lipin-1 also acts as a transcriptional regulator in the nucleus as well. Lipin-1 contains a putative nuclear localization signal (NLS) and the lipin-1α isoform is localized in the nucleus of liver cells [25] and mature adipocytes [14]. In yeast, the homolog of lipin (Pah1p/Smp2p) associates with the promoter region of genes involved in phospholipid biosynthesis and regulates the expression of these genes [26]. In mammalian cells, lipin-1 can interact with nuclear receptors such as PPARα, PPARγ, hepatocyte nuclear factor 4α, and glucocorticoid receptor [15]. Furthermore, lipin-1 promotes PPARα-dependent gene expression in mouse liver by mediating recruitment of PGC-1α and p300 [15]. Recently, it has been suggested that lipin-1 also may function as a transcriptional coactivator during adipogenesis [27] and exercise-induced mitochondrial biogenesis [28]. Up to now, it remains to be determined whether lipin-1 functions in the nucleus in neuronal cells.

In this study, we show that ectopically expressed lipin-1α is localized in both the cytoplasm and the nucleus in neuronal cells, and that its nuclear localization is controlled by sumoylation occurring at two consensus sites. We are able to detect transcriptional coactivator effects of ectopic lipin-1α in neuronal cells, and show that sumoylation is required for this activity, consistent with the effects of sumoylation on the nuclear localization of lipin-1α. Since we find that lipin-1 is expressed and sumoylated endogenously in mouse brain, our work on cultured neuronal cells raises the possibility that lipin-1 has transcriptional functions in the brain that are controlled by sumoylation.

## Results

**Figure 1:**
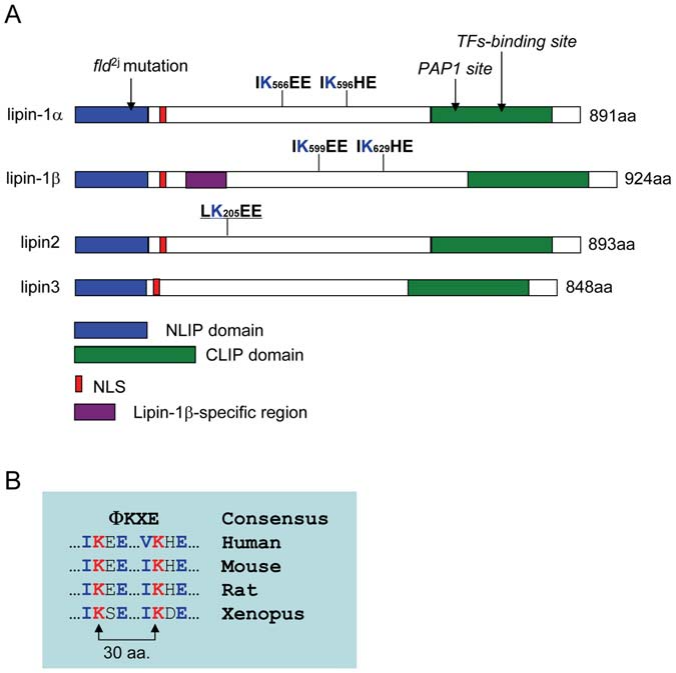
Sequence organization and sumoylation motifs in lipins. (A) A schematic depicting the homologous regions in lipin-1α, lipin-1β, lipin-2 and lipin-3, and the ΦKXE sumoylation motifs in lipin-1α, lipin-1β, and lipin-2. The N-terminal lipin-homology domain (NLIP) (blue), C-terminal lipin-homology domain (CLIP) (green), and the predicted nuclear localization signal (NLS) (red) are indicated. Shown also is the relative location of the lipin-1β-specific insert (purple). (B) Conservation of the ΦKXE sumoylation motifs of lipin-1 from various species.

**Figure 2:**
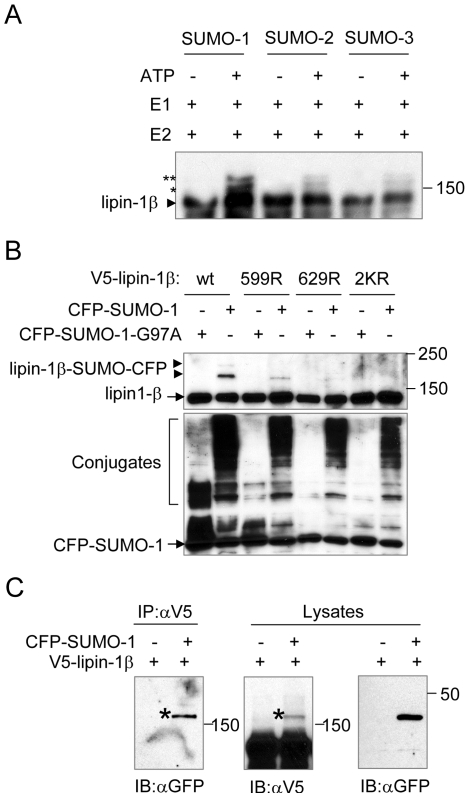
Lipin-1β is sumoylated at two consensus sumoylation sites. (A) Immunopurified V5-tagged lipin1β was incubated with or without recombinant SUMO-1, 2, or 3, the E1 enzyme Aos1/Uba2 and the E2-conjugating enzyme Ubc9 as indicated. The reaction mixture then was analyzed by immunoblotting with anti-V5 antibody. Asterisks indicate forms of lipin1β (arrowhead) that are modified with one or two SUMO molecules. (B) HeLa cells were cotransfected with V5-lipin-1β together with CFP-SUMO-1 or CFP-SUMO-1-G97A as indicated. 48 h after transfection, the cells were directly lysed in Laemmli sample buffer supplemented with 10 mM NEM and analyzed by immunoblotting (IB) with anti-V5 (upper panel) or anti-GFP (lower panel) antibody. In the upper panel, the arrowheads indicate the slower migrating forms of lipin-1β. In the lower panel, the free CFP-SUMO1 and the endogenous proteins conjugated with CFP-SUMO-1, respectively, are indicated. (C) Lysates of HeLa cells expressing V5-tagged lipin-1β and CFP-tagged SUMO-1 were subjected to immunoprecipitation (IP) with anti-V5 antibody, followed by immunoblotting (IB) with anti-GFP to detect sumoylated lipin-1β. Asterisks denote the sumoylated form of lipin-1β.

To determine whether the mammalian lipins are potential targets for sumoylation, we examined the amino acid sequence of lipin-1 (including lipin-1α and lipin-1β), lipin-2 and lipin-3. This analysis revealed that both lipin-1α and lipin-1β contains two sumoylation consensus motifs, and lipin-2 contains one (Figs. 1A and 1B). We first evaluated whether lipin-1β, the longer isoform of lipin-1, can be sumoylated *in vitro*. Immunopurified V5-tagged lipin-1β was incubated with the SUMO E1 activating enzyme Aos1/Uba2, the E2 conjugating enzyme Ubc9, and either SUMO-1, SUMO-2 or SUMO-3, in the presence or absence of ATP. Subsequent analysis of this reaction by immunoblotting with anti-V5 antibody revealed the generation of two bands that migrated more slowly than lipin-1β in the samples that contained each of the three SUMO paralogs. The bands were strongest in the sample incubated with SUMO-1, as compared to SUMO-2 or SUMO-3 (Fig. 2A). The sizes of these products suggested that they represented forms of lipin-1β that are modified with one or two SUMO proteins (Fig. 2A). When ATP was absent from the reaction, these bands were not detected (Fig. 2A), consistent with the requirement of energy for SUMO conjugation [2]. These results indicate that lipin-1β can be modified by SUMO-1, SUMO-2, and SUMO-3 *in vitro*.

To examine whether lipin-1β can be sumoylated *in vivo*, HeLa cells were co-transfected with plasmids encoding V5-tagged lipin-1β and CFP-fused SUMO-1. Immunoblot analysis of extracts from these cells with an anti-V5 antibody revealed a strong band corresponding to V5-lipin-1β as well as a weaker, more slowly migrating band at the mobility expected for V5-lipin-1β modified with one molecule of CFP-SUMO-1 (Fig. 2B, lower arrowhead). A form of lipin-1β that might be modified with two molecules of CFP-SUMO-1 also was faintly detected (Fig. 2B, upper arrowhead). To investigate whether these bands represent V5-lipin-1β covalently conjugated to CFP-SUMO-1, a CFP-SUMO-1 mutant (G97A) that cannot be conjugated to target proteins [29] was used instead of the wild-type CFP-SUMO-1. In this case, both additional bands were absent (Fig. 2B). As a separate approach to confirm that lipin-1β is modified by CFP-SUMO-1 in HeLa cells, a lysate from the transfected cells was immunoprecipitated with an anti-V5 antibody, and the immunoprecipitate was examined by immunoblotting with an anti-GFP antibody. This analysis revealed an anti-GFP reactive band in the immunoprecipitate that comigrated with the form of V5-lipin-1β presumptively modified with one molecule of SUMO-1 (Fig. 2C). Together, these experiments indicate that lipin-1β is sumoylated in HeLa cells *in vivo*.

We next examined whether K599 or K629 of lipin-1β, each of which are located within the sumoylation consensus sequence ΦKXE, are the sites of sumoylation. As shown in Fig. 2B, mutation of either K599 or K629 alone substantially reduced sumoylation of lipin-1β. However, simultaneous mutation of the two lysines completely abrogated the sumoylation of lipin-1β *in vivo*. Furthermore, we found that the immunopurified V5-lipin-1β mutant (K599/626R, “2KR”) was not modified by SUMO-1 in an *in vitro* sumoylation reaction (data not shown). This indicates that lipin-1β can be sumoylated at either of the sumoylation consensus sites. Although a form of lipin-1β that was modified with two molecules of CFP-SUMO-1 was clearly seen by *in vitro* sumoylation of lipin-1β using untagged SUMO-1 (Fig. 2A), this form was scarcely detectable in the HeLa cell assay (Fig. 2B). This discrepancy might be a consequence of steric interference by CFP in the latter case, which could diminish modification of the second lysine when the first lysine is sumoylated.

**Figure 3:**
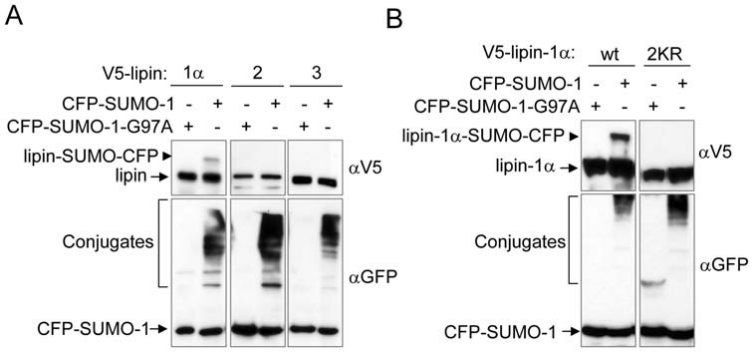
Lipin-1α, but not lipin-2 and lipin-3 is sumoylated in HeLa cells. (A and B) HeLa cells were cotransfected with CFP-SUMO-1 or CFP-SUMO-1-G97A together with V5-lipin-1α, V5-lipin-2 or V5-lipin-3 (A) or with V5-lipin-1α-2KR (B) as indicated. 48 h after transfection, the cells were directly lysed in Laemmli sample buffer supplemented with 10 mM NEM and analyzed by immunoblotting with anti-V5 (upper panel) or anti-GFP (lower panel) antibody. In the upper panels, the arrowheads indicate the slower migrating form of lipin. In the lower panel, the free CFP-SUMO1 and the endogenous proteins conjugated with CFP-SUMO-1, respectively, are indicated. The levels of CFP-SUMO-conjugates varied due to different exposures.

Next, we examined whether lipin-1α, the shorter splice isoform of lipin-1 that lacks a 33 amino acid adjacent to the nuclear localization sequence (NLS) (Fig. 1A), can be sumoylated *in vivo*. As shown in Fig. 3A, a band corresponding to lipin-1α modified with one molecule of SUMO-1 was obtained when HeLa cells were transfected with expression plasmids for V5-lipin-1α and CFP-SUMO-1. A presumptive minor form of V5-lipin-1α with two molecules of CFP-SUMO-1 is seen upon long exposure (Fig. S1). The bands were not obtained when the mutant CFP-SUMO1-G97A was used (Figs. 3A and S1). Furthermore, simultaneous mutation of the two putative sumoylation sites (K566/596R, “2KR”) also blocked lipin-1α sumoylation, similar to lipin-1β (Fig. 3B and Fig. S1). However, both the G84R and D679E mutations in lipin-1α that are linked to lipodystrophy [18] and deficiency in PAP activity [15], [30], respectively, did not lead to a diminished sumoylation of lipin-1α (Fig. S1). In contract to the results obtained with lipin-1, we did not detect sumoylation of lipin-2 and lipin-3 in transfected HeLa cells (Fig. 3A), even though lipin-2 bears a sumoylation consensus site (Fig. 1A). It is conceivable that the potential sumoylation site of lipin-2 is inaccessible in its 3-D structure and is never modified, or that sumoylation of lipin-2 occurs only in a specific cellular context.

**Figure 4:**
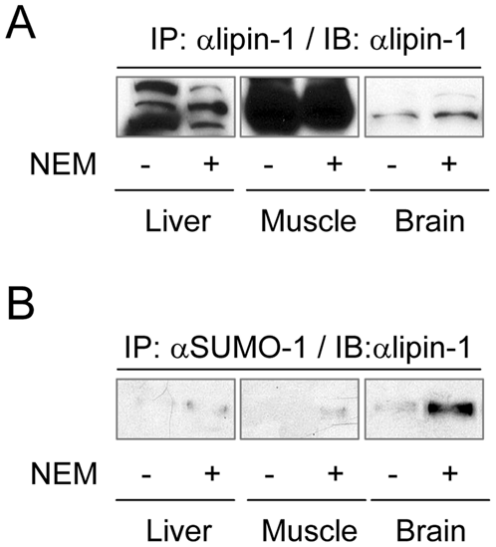
Sumoylation of lipin-1 in mouse tissues. Tissue samples were prepared in lysis buffer with or without NEM. After determination of protein concentrations, 1 mg of tissue extracts were immunoprecipitated with anti-lipin-1 (A) or anti-SUMO-1 (B). The immunoprecipitates were analyzed by immunoblotting with anti-lipin-1 antibody.

To investigate whether endogenous lipin-1 is sumoylated, we examined mouse liver, muscle and brain, three of the tissues where lipin-1 mRNA is expressed [31]. The tissues were lysed in the absence or presence of N-ethylmaleimide (NEM), which inhibits SUMO-specific proteases, and then were immunoprecipitated with an anti-lipin-1 or anti-SUMO-1 antibody. Immunoblot analysis of samples immunoprecipitated with anti-lipin-1 showed that lipin-1 protein was present in all three tissues, albeit at much higher levels in muscle than in the other two tissues (Fig. 4A). Our results on the tissue expression of lipin-1 protein are consistent with the relative mRNA levels reported for lipin-1 in these tissues [31]. The multiple bands appearing in the liver sample presumably result from specific post-translational modifications of lipin-1 occurring in this tissue (e.g. phosphorylation [30], [32]) (Fig. 4A). Immunoprecipitation with anti-SUMO-1 followed by immunoblotting with anti-lipin-1 revealed a prominent sumoylated form of lipin-1 in brain, but only when the tissue extracts were prepared in the presence of NEM (Fig. 4B). Sumoylated forms of lipin-1 in muscle and liver were only marginally detected (Fig. 4B). These results suggest that sumoylation of lipin-1 may be particularly important for its functions in brain. Thus, we focused our subsequent analysis on neuronal cells.

**Figure 5:**
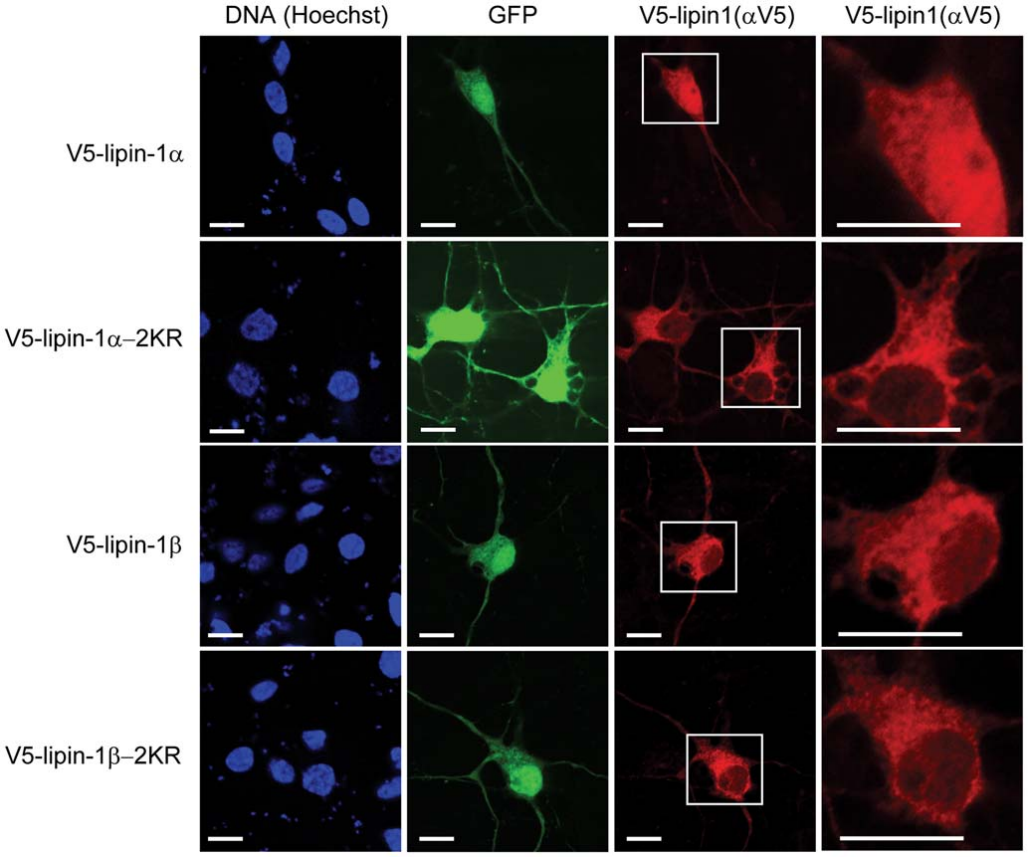
Localization of lipin-1 isoforms and sumoylation site mutants in rat embryonic cortical neurons. Cortical neurons were cotransfected with GFP and the indicated plasmids. 48 h later, the localization of the V5-tagged proteins (red) was determined by immunofluorescence with anti-V5 antibody. Nuclei (blue) were visualized with Hoechst 33342 staining. GFP fluorescence (green) was also captured. The magnified images for V5-lipin-1 immunostaning were also provided to more clearly show the cytosolic distribution of lipin-1α, 1β, and their mutants. Bar, 10 µm.

Because sumoylation is known to regulate the nuclear localization of some of its target proteins, we examined whether sumoylation of lipin-1 affects its subcellular distribution in primary neurons. Cerebrocortical neurons from embryonic day 17 rat embryos were isolated and cultured for 3 days *in vitro*. Cells then were transfected either with V5-tagged lipin-1α or lipin-1β, or with the corresponding double sumoylation site mutants, and two days later the subcellular distribution of the lipin-1 isoforms were detected by immunofluorescence with anti-V5 antibody. As shown in Fig. 5, ectopic lipin-1α exhibited both nuclear and cytoplasmic localization in embryonic cortical neurons, with a higher concentration in the nucleus. In contrast, lipin-1β showed exclusively cytoplasmic localization and was excluded from the nucleus. Interestingly, the sumoylation site lipin-1α-2KR mutant almost completely lost its nuclear localization compared to wild-type lipin-1α, although its cytoplasmic localization remained unchanged. Also, the almost exclusively cytoplasmic localization of lipin-1β did not change with its sumoylation site mutant (Fig. 5).

**Figure 6:**
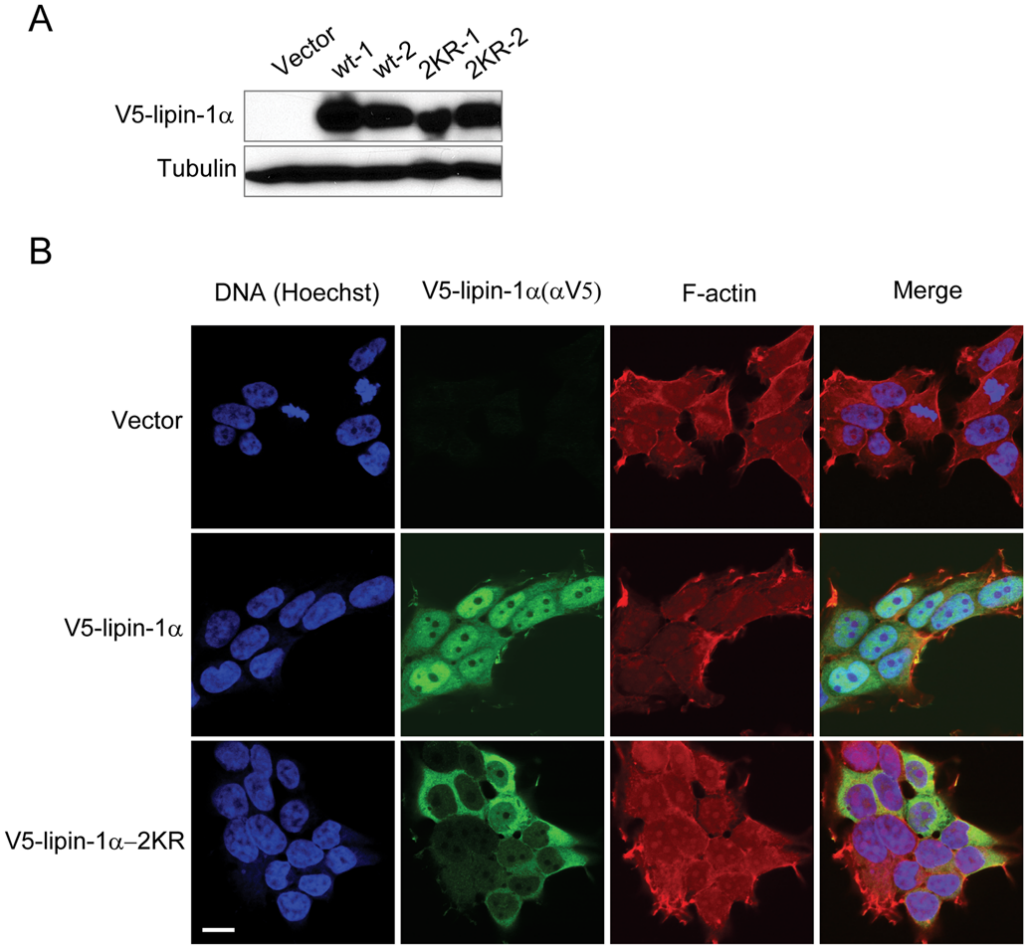
Localization of lipin-1α and lipin-1α-2KR in stably transfected SH-SY5Y cells. (A) Immunoblot analysis of V5-lipin-1α and V5-lipin-1α-2KR in stably transfected SH-SY5Y cell clones. Shown is analysis of two separate V5-lipin-1α clones (wt-1 and wt-2), and two separate V5-lipin-1α-2KR clones (2KR-1 and 2KR-2). (B) SH-SY5Y cells stably transfected with V5-lipin-1α (wt-1) or V5-lipin-1α-2KR (2KR-1) were subjected to immunostaining with anti-V5 antibody (green). F-actin (red) and nucleus (blue) were stained with Alex598-Phalloidin and Hoechst 33342, respectively. Bar, 10 µm.

To extend the observations obtained with cortical neurons, we generated SH-SY5Y neuronal cell lines stably expressing V5-lipin-1α or V5-lipin-1α-2KR. Two clones were examined for each transfectant to control for potential non-specific effects of stable chromosomal integration. Immunoblot analysis revealed that the V5-lipin-1α and V5-lipin-1α-2KR were expressed at comparable levels in these clones (Fig. 6A). Employing immunofluorescence analysis of these SH-SY5Y clones, we observed that ectopic lipin-1α exhibited both nuclear and cytosolic localization, with a higher concentration in the nucleus (Fig. 6B). Conversely, lipin-1α-2KR showed almost exclusively cytoplasmic localization (Fig. 6B), similar to our findings in cortical neurons (Fig. 5). Identical results were obtained with both of the V5-lipin-1α and V5-lipin-1α-2KR stable clones (data not shown). We confirmed that in both cortical neurons and SH-SY5Y cells, the lipin-1α was sumoylated at the two consensus sites (Fig. S2). Interestingly, the sumoylation-dependent nuclear localization of lipin-1α appears to occur in a cell type-specific manner. Whereas sumoylation facilitated nuclear distribution of lipin-1α in HeLa cells, as judged by the analysis of lipin-1α and the lipin-1α-2KR mutants, both of these forms of lipin-1α were concentrated in the cytoplasm in HEK293A (Fig. S3), thus suggesting a cell-type-specific cytosolic/nuclear retention mechanism.

**Figure 7:**
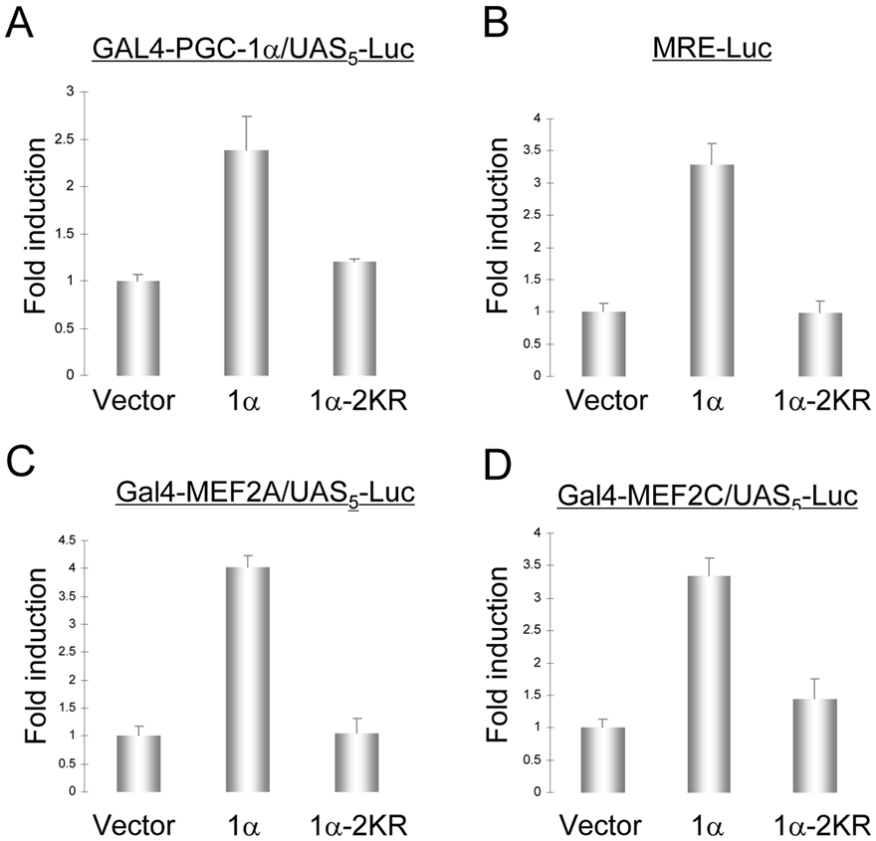
Effect of lipin-1α and lipin-1α-2KR on coactivation of PGC-1α and MEF2 in SH-SY5Y cells. SH-SY5Y clones stably expressing V5-lipin-1α or V5-lipin-1α-2KR were transfected with Gal4-PGC-1α and (UAS)_5_-TATA-Luc (A), MRE-Luc (B), Gal4-MEF2A and (UAS)_5_-TATA-Luc (C), or Gal4-MEF2C and (UAS)_5_-TATA-Luc (D). 36 h later, the relative luciferase activity was determined. Error bars indicate standard deviations from 4 independent samples.

The presence of lipin-1α in both the nucleus and the cytoplasm of neuronal cells suggests that it may be a nucleocytoplasmic shuttling protein. Since nuclear accumulation is required for a nucleocytosolic shuttling protein to function in regulation of gene expression, we examined whether sumoylation can regulate the activity of lipin-1α as a transcriptional coactivator. We first examined effects on lipin-1α stimulation of PGC-1α activity, which was originally described in liver [15]. For this purpose, SH-SY5Y neuronal cells that stably overexpressed lipin-1α or lipin-1α-2KR were transfected with a vector encoding a GAL4-PGC-1α fusion protein together with the corresponding luciferase reporter plasmid. As shown in Fig. 7A, the transactivation mediated by the PGC-1α fusion protein was significantly enhanced in cells expressing lipin-1α, but not in cells expressing lipin-1α-2KR. Thus, lipin-1α can stimulate the transcriptional coactivation function of PGC-1α in cultured neuronal cells in a sumoylation-dependent manner.

We next examined a possible effect of lipin-1α and its sumoyation on the transcriptional activity of MEF2, a transcription factor that regulates neuronal survival and differentiation [33]-[36]. We found that SH-SY5Y cells overexpressing lipin-1α showed marked stimulation of the transcriptional activity of endogenous MEF2, using an MEF2-responsive luciferase plasmid as a reporter (Fig. 7B). We also observed that overexpressed lipin-1α stimulated the transactivation of two separate MEF2 isoforms (MEF2A and MEF2D), using a Gal4-MEF2 fusion transactivation system (Figs. 7C and D). The possibility that lipin-1α stimulates the transcriptional activity of MEF2 by a direct coactivator mechanism is supported by our finding that lipin-1α coimmunoprecipitated with endogenous MEF2C in mouse brain extract (Fig. S4A) and with ectopically expressed MEF2C in HEK293A cells (Fig. S4B). As predicted, the lipin-1α mutant that was deficient in nuclear localization also showed a defect in stimulation of MEF2 transcription (Figs. 7B-7D). Thus, these data suggest that sumoylated lipin-1α is a transcriptional coactivator for MEF2 as well as for PGC-1α, as demonstrated previously.

The defect of lipin-1α-2KR in transcriptional coactivation apparently was not due to a loss of PAP1 activity, as immunopurified lipin1α-2KR obtained from SH-SY5Y cells still retained Mg^2+^-dependent phosphatase activity for PA (C8), but not for LPA (C18:1), determined with the malachite green assay (Fig. S5). In contrast, immunopurified V5-LPP3 (a PAP2 family member) was able to dephosphorylate both PA (C8) and LPA (C18:1) in a Mg^2+^-independent manner (data not shown). Lipin-1α-2KR also retained the ability to associate with both PGC-1α and MEF2C, as determined by coimmunoprecipitation from co-transfected HEK293A cells (Figs. S4B and S4C), where both lipin-1α and lipin-1α-2KR were cytoplasmically localized (Fig. S3). Together, these observations suggest that sumoylation regulates the nuclear localization and the coactivator activity of lipin-1α in neuronal cells.

## Discussion

Lipin-1 has important functions as both a PAP and a transcriptional coactivator, but relatively little is known about post-translational regulation of its functions. The only previously characterized posttranslational modification of lipin-1 is phosphorylation, which can regulate its localization to the ER membrane vs cytosol [30], and its PAP activity during the cell cycle [32]. Also, the transcriptional coactivator function of the yeast homolog of lipin-1, Pah1p, is regulated by the phosphatase Nem1p [26]. However, it remains unknown how the mammalian homolog of yeast Nem1p, the nuclear envelope protein Dullard/NET56 [37], [38] might affect nuclear functions of mammalian lipin-1.

Our current study reveals for the first time that lipin-1α and lipin-1β undergo sumoylation *in vivo*. Although most of our work involved analysis of lipin-1 that was ectopically expressed in cultured cells, we also demonstrated that endogenous lipin-1 in rodent brain is sumoylated. This indicates that sumoylation of lipin-1 is physiologically relevant. In the cultured cell models we examined, lipin-1 sumoylation occurs on the two consensus sumoylation sites present in both lipin-1α and lipin-1β. We found that lipin-1α, which is the predominant lipin-1 isoform in the brain [14], is concentrated in the nucleus in both embryonic cortical neurons and cultured SH-SY5Y neuronal cells, and that its nuclear localization is dependent on the presence of functional sumoylation sites. In contrast, lipin-1β is mostly excluded from the nucleus in the neuronal cells we examined.

Our observations on the different nucleocytoplasmic localizations of lipin-1α and lipin-1β in neuronal cells are reminiscent of previous findings in mature adipocytes, where lipin-1α and lipin-1β were found to be localized to the nucleus and to the cytoplasm, respectively [14]. Although we do not yet know how sumoylation facilitates the nuclear localization of lipin-1α in neuronal cells, sumoylation might influence its nuclear localization by controlling interactions with the nuclear import/export machinery, and/or by regulating its binding to intranuclear components. In this regard, a recent survey of proteins whose nuclear localization is facilitated by sumoylation reveals that sumoylation sites are frequently located in close proximity to NESs [13]. In these cases, sumoylation might affect nuclear export by inhibiting the binding of NESs to export receptors. The relevance of this mechanism to nuclear export of lipin-1α remains to be determined.

Sumoylation of lipin-1α, in addition to mediating its nuclear localization, also appears to be required for its transcriptional coactivator activity toward PGC-1α and MEF2 in neuronal cells (see paragraph below). This transcription-related effect of sumoylation most likely is a simple consequence of its ability to mediate the nuclear localization of lipin-1α, since the sumoylation site mutant of lipin-1α still retains the ability to associate with PGC-1α and MEF2 in cultured cells. We also determined that sumoylation does not influence the PAP1 activity of lipin-1α. This is consistent with the notion that the lipin homologs in yeast and in invertebrates bears no consensus sumoylation site, even though lipin has PAP1 activity in all eukaryotes examined.

Although lipin-1 is known to be involved in regulation of gene expression in liver and adipocytes, its functional relevance to gene expression in neuronal cells is unknown. Our observations that lipin-1α shows both nuclear localization and transcriptional coactivator activity towards PGC-1α in neuronal cells, suggest that it indeed may act as a transcriptional coactivator in brain. Furthermore, our results argue that sumoylation of lipin-1α is likely to be important for these putative brain functions. PGC-1α was recently found to have major functions in brain, since it controls the expression of a subgroup of antioxidant genes [39], and its dysfunction is associated with neurodegenerative diseases [40]–[42]. Our findings, therefore, may provide new clue to understand the regulation of PGC-1α in brain.

Whereas the ability of lipin-1 to promote the coactivation of genes by PGC-1α has been previously established [15], our results for the first time indicate that lipin-1α also can associate with and coactivate MEF2, a critical transcription factor in control of neuron survival and differentiation [35], [36]. However, it remains to be determined whether this coactivation occurs at the level of MEF2-associated promoters, and which of the MEF2-target genes are regulated by lipin-1α. It should be pointed out that PGC-1α can stimulate its own promoter by coactivating MEF2 [43]. Moreover, PGC-1α can positively regulate lipin-1 expression [15]. In this regard, lipin-1α may serve as an amplifier of PGC-1α-MEF2 loop in neuronal cells, using sumoylation as a molecular switch.

In summary, we have shown that sumoylation can serve as a molecular switch to control the nuclear localization and coactivator activity of lipin-1α in cultured neuronal cells. Our results raise the possibility that lipin-1α acts as a sumoylation-regulated transcriptional coactivator in brain; however, the effects of lipin-1β sumoylation remain to be determined. Identifying the functions of lipin-1 sumoylation in brain (e.g. which genes are transcriptionally controlled by this mechanism and whether this is linked to neuron survival or differentiation) and revealing its physiological and pathological relevance (e.g. whether this is involved in regulation of learning and memory and/or implicated in neuropathy) represent important challenges in future studies.

## Materials and Methods

### Chemicals, plasmids and antibodies

Phosphatidic acid (PA: C8), and lysophosphatidic acid (LPA: C18:1) were purchased from Avanti Polar Lipids. N-ethylmaleimide (NEM) was purchased from Sigma. V5-tagged lipin-1α, lipin-1β, lipin-2 and lipin-3 expression vectors were kind gifts from Dr. Karen Reue (University of California, Los Angeles) [14], [31]; CFP-SUMO-1 and CFP-SUMO-1(G97A) were generously provided by Dr. Takashi Osumi (University of Hyogo, Japan) [29]; Gal4-MEF2A and Gal4-MEF2C were kindly provided by Hung-Ying Kao (Case Western Reserve University and the Research Institute of University Hospitals of Cleveland) [44]; Gal4-PGC-1α and Myc-PGC-1α were gifts from Daniel Kelly (Washington University School of Medicine) [15]; (UAS)_5_-TATA-Luc was described previously [45], [46]. All the plasmid-based mutations employed in this study were created using GeneTailor™ site-directed mutagenesis system (Invitrogen) according to the manufacturer's protocol. The rabbit anti-mouse lipin-1 antibody was kindly provided by Thurl E. Harris (University of Virginia) [22], [30]. The following antibodies were obtained commercially: anti-V5 (Invitrogen); anti-Myc (Santa Cruz Biotechnology, Inc); anti-lipin-1 (Chemicon); anti-alpha-tubulin (Abcam); anti-MEF2C (Santa Cruz Biotechnology, Inc); anti-GFP (Santa Cruz Biotechnology, Inc); anti-Gal4 (Santa Cruz Biotechnology, Inc); anti-V5 agarose (Sigma); anti-SUMO1 agarose (Santa Cruz Biotechnology, Inc); Alexa Fluor488™ or Alexa Fluor568™ anti-IgG antibody (Invitrogen); HRP-conjugated anti-IgG antibody (Pierce).

### Cell culture and transfection

HeLa and SH-SY5Y cells were purchased from ATCC, HEK293A cells were purchased from Invitrogen. They were all maintained in DMEM supplemented with 10% fetal bovine serum (FBS) (Invitrogen) and antibiotics. Rat cortical neuron cultures were prepared as described previously [47]. Cell transfections were performed with Lipofectamine™ 2000 (Invitrogen). Stable SH-SY5Y populations were generated by selection with 400 µg/ml G418 for 3 weeks and 2 independent clones were chosen. The clones were confirmed by immunostaining with anti-V5 antibody, which revealed that 100% of the cells were positive for V5-lipin-1α or V5-lipin-1α-2KR.

### Immunoprecipitation and immunoblotting

Cells were harvested 48 h after transfection and lysed by brief sonication in lysis buffer (50 mM Tris pH 7.5, 250 mM NaCl, 1 mM EDTA, 1 mM EGTA, 0.5% Triton X-100, 10% glycerol, complete Protease Inhibitor Cocktail (Roche Diagnostics)). Lysates were subjected to immunoprecipitation with anti-V5 agarose. Immune complexes were washed five times with lysis buffer and subjected to immunoblotting with anti-GFP, anti-Gal4, or anti-Myc antibody.

### *In vitro* and *in vivo* sumoylation

For *in vitro* sumoylation experiments, HEK293A cells were transfected with plasmids expressing V5-tagged proteins. 48 h later, lysates were subjected to immunoprecipitation with anti-V5 agarose, and immune complexes were eluted with V5-peptide (100 µg/ml). The immunopurified proteins were incubated with 2 µg of recombinant SUMO-1 (BIOMOL), 0.5 µg of Aos1/Uba2 (BIOMOL International), and 0.1 µg of Ubc9 (BIOMOL) for 2 h in 20 µl of 50 mM Tris-HCl pH 7.5, 5 mM MgCl_2_, 2 mM ATP. The reaction mixture was analyzed by immunoblotting with anti-V5 antibody. *In vivo* sumoylation analysis was performed as previously described [48]. In brief, HeLa cells were transfected with expression plasmids for V5-lipin-1β and CFP-SUMO-1 (or their mutants). 48 h after transfection, cells were directly lysed in Laemmli SDS gel buffer supplemented with 10 mM NEM. Extracts were boiled for 10 min prior to loading on SDS-polyacrylamide gels. To detect sumoylation of endogenous lipin-1, tissue samples were cut into small pieces in ice-cold PBS containing 10 mM NEM, lysed by douncing and subsequent sonication in ice-cold lysis buffer (50 mM Tris pH 7.5, 250 mM NaCl, 1 mM EDTA, 1 mM EGTA, 10 mM NEM, 0.5% Triton X-100, 10% glycerol, complete Protease Inhibitor Cocktail (Roche Diagnostics)). The lysates were incubated with anti-SUMO-1 agarose or lipin-1 antibody followed by protein A/G PLUS (Santa Cruz Biotechnology, Inc) with agitation overnight. Immune complexes were analyzed by immunoblotting with anti-lipin-1 antibody.

### Immunofluorescence microscopy

Immunofluorescent staining was performed as previously described [49]. In brief, cells were fixed with 4% formaldehyde in PBS for 30 min. Following fixation, cells were treated with 0.2% Triton X-100 in PBS for 5 min. After blocking with 10% FBS in PBS for 20 min, cells were incubated for 1 h with primary antibody, followed by washing in PBS and incubation for 1 h with the relevant secondary antibody. F-actin and nuclei were stained with Alex598-Phalloidin and Hoechst 33342 (Invitrogen), respectively. The stained cells on cover slips were subsequently mounted in Gel Mount™ (Biomeda) and sealed with Clarion™ (Biomeda). Cell images were taken with the BioRad-Zeiss Radiance 2100 laser scanning confocal microscope and analyzed by LaserSharp 2000™ software (Bio-Rad). Images were pseudo-colorized and merged with ImageJ.

### Lucifease assay

The luciferase assay was performed as previously described [45], [46].

### PAP1 activity assay

SH-SY5Y cells stably expressing pcDNA3, lipin-1α-V5 or lipin-1α-2KR-V5 were lysed by scraping into ice cold lysis buffer (50 mM Tris pH 7.5, 250 mM NaCl, 1 mM EDTA, 1 mM EGTA, 0.5% Triton X-100, 10% glycerol, complete Protease Inhibitor Cocktail (Roche Diagnostics)). Samples were sonicated on ice and centrifuged at 16,000 g at 4°C for 30 min. The supernatant was mixed with anti-V5 agarose and rotated at 4°C overnight. The beads were washed with lysis buffer 4 times, and with the assay buffer (100 mM Tris-maleate, pH 6.5, 0.5% Triton X-100, 10 mM DTT) twice. The recombinant lipin-1α-V5 or lipin-1α-2KR-V5 was eluted by the incubation with 70 µl V5-peptide (100 µg/ml) in assay buffer for 1 h at 4°C with agitation. After brief centrifugation, the supernatants were subjected to either immunoblotting with anti-V5 antibody or analysis of PAP1 activity using the malachite green colorimetric assay as described previously [50], [51], with minor modifications. In brief, the assays were performed in 96-well, clear, non-binding surface assay plates (Corning Incorporated) in 40 µl of assay buffer containing either PA or LPA at 100 µM in the absence or the presence of 5 mM MgCl_2_ and 10 µl of immuno-purified lipin-1α-V5 or lipin-1α-2KR-V5. The reaction was carried out at 37°C for 90 min and stopped with 100 µl of malachite green reagent (BIOMOL), and the absorbance was read at 650 nm according to the manufacturer's instructions. The enzyme reactions were linear over time and protein concentration.

## Supporting Information

Figure S1 Sumoylation of various lipin-1α mutants in HeLa cells. Cells were cotransfected with V5-lipin-1α or the indicated lipin1 mutants, together with CFP-SUMO-1 or CFP-SUMO-1-G97A, as indicated. 48 h after transfection, the cell lysates were lysed in Laemmli sample buffer supplemented with 10 mM NEM and analyzed by immunoblotting (IB) with anti-V5 (upper panel) or anti-GFP (lower panel) antibody. In the upper panels, the arrowheads indicate the slower migrating forms of lipin-1α. In the lower panel, the free CFP-SUMO1 and the endogenous proteins conjugated with CFP-SUMO-1, respectively, are indicated.
(0.43 MB TIF)

Figure S2 Sumoylation of lipin-1α and lipin-1α-2KR in neuronal cells. SH-SY5Y cells (A) or cortical neurons (B) were cotransfected with V5-lipin-1α or V5-lipin-1α-2KR, together with CFP-SUMO-1 or CFP-SUMO-1-G97A as indicated. 48 h after transfection, the cells were directly lysed in Laemmli sample buffer supplemented with 10 mM NEM and analyzed by immunoblotting with anti-V5 antibody. The arrowheads indicate the slower migrating form of lipin-1α.
(0.12 MB TIF)

Figure S3 Localization of lipin-1α and lipin-1α-2KR in HEK293A and HeLa cells. HEK293A cells (A) or HeLa cells (B) were transfected with lipin-1α and lipin-1α-2KR. 48 h later, the cells were subjected to immunostaining with anti-V5 antibody (green). F-actin (red) and nucleus (blue) were stained with Alex598-Phalloidin and Hoechst 33342, respectively. Bar, 10 µm. (C) The distribution patterns of V5-lipin-1α, V5-lipin-1β, or their mutants were scored for almost 100 cells and classified into three categories: C>N, cytoplasmic-dominant distribution; N = C, roughly equal distribution in nuclear and cytoplasmic compartments; and C<N, nuclear-dominant distribution.
(1.03 MB TIF)

Figure S4 Sumoylation site lipin-1α mutant retains the capacity to associate with MEF2C and PGC-1α. (A) Mouse brain extract was prepared and immunoprecipitated with anti-lipin-1 antibody or a control IgG, and the MEF2C present in the immunoprecipitates was analyzed by immnoblotting. (B, C) HEK293A cells were transfected with V5-lipin-1α or V5-lipin-1α-2KR, together with Gal4-MEF2C (B) or Myc-PGC-1α (C). 48 h later, the cell lysates were subjected to immunoprecipitation with anti-V5 agarose, and the presence of Gal4-MEF2C (B) or Myc-PGC-1α (C) in the immunoprecipitates was analyzed by immunoblotting with anti-Gal4 or anti-Myc antibody.
(0.33 MB TIF)

Figure S5 Lipin-1α and lipin-1α-2KR have comparable PAP1 activity in stably transfected SH-SY5Y cells. (A) V5-tagged lipin-1α or lipin-1α-2KR was immunopurified from SH-SY5Y cells and then subjected to the PAP1 activity analysis using PA as the substrate. Bottom: The expression of immunopurified lipin-1α or lipin-1α-2KR was determined by immunoblotting with anti-V5 antibody. (B) Immunopurified lipin-1α was subjected to the PAP1 activity analysis using PA or LPA as the substrate, in the presence or absence of Mg^2+^.
(0.12 MB TIF)
