# Supplementary figures and images for: Sumoylation Regulates Nuclear Localization of Lipin-1α in Neuronal Cells
Source: PLoS One. 2009 Sep 15;4(9):e7031. doi: 10.1371/journal.pone.0007031 (PMC2737637; doi:10.1371/journal.pone.0007031)

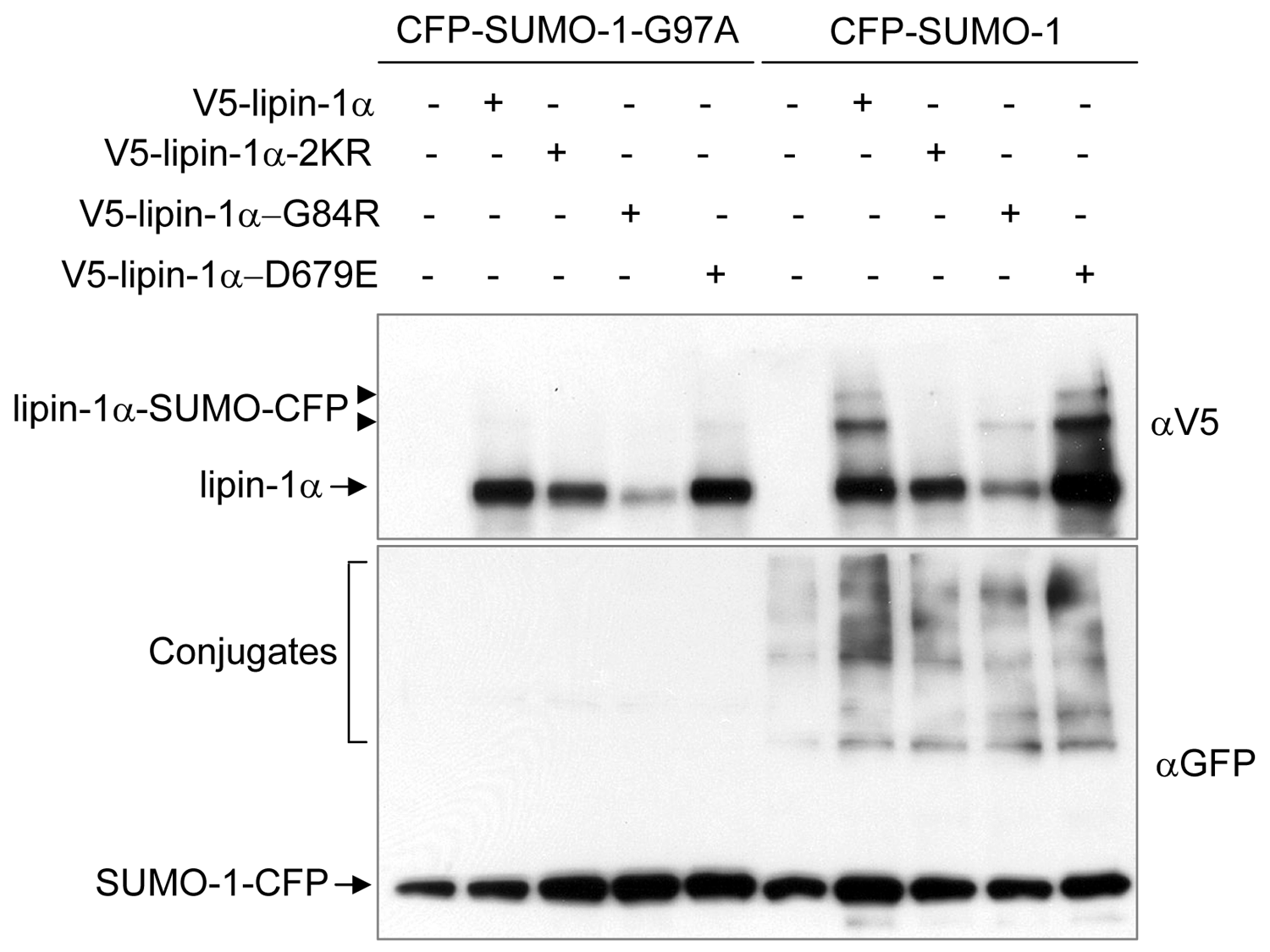

Supplement: Figure S1 — Sumoylation of various lipin-1α mutants in HeLa cells. Cells were cotransfected with V5-lipin-1α or the indicated lipin1 mutants, together with CFP-SUMO-1 or CFP-SUMO-1-G97A, as indicated. 48 h after transfection, the cell lysates were lysed in Laemmli sample buffer supplemented with 10 mM NEM and analyzed by immunoblotting (IB) with anti-V5 (upper panel) or anti-GFP (lower panel) antibody. In the upper panels, the arrowheads indicate the slower migrating forms of lipin-1α. In the lower panel, the free CFP-SUMO1 and the endogenous proteins conjugated with CFP-SUMO-1, respectively, are indicated. (0.43 MB TIF) [file pone.0007031.s001.tif]

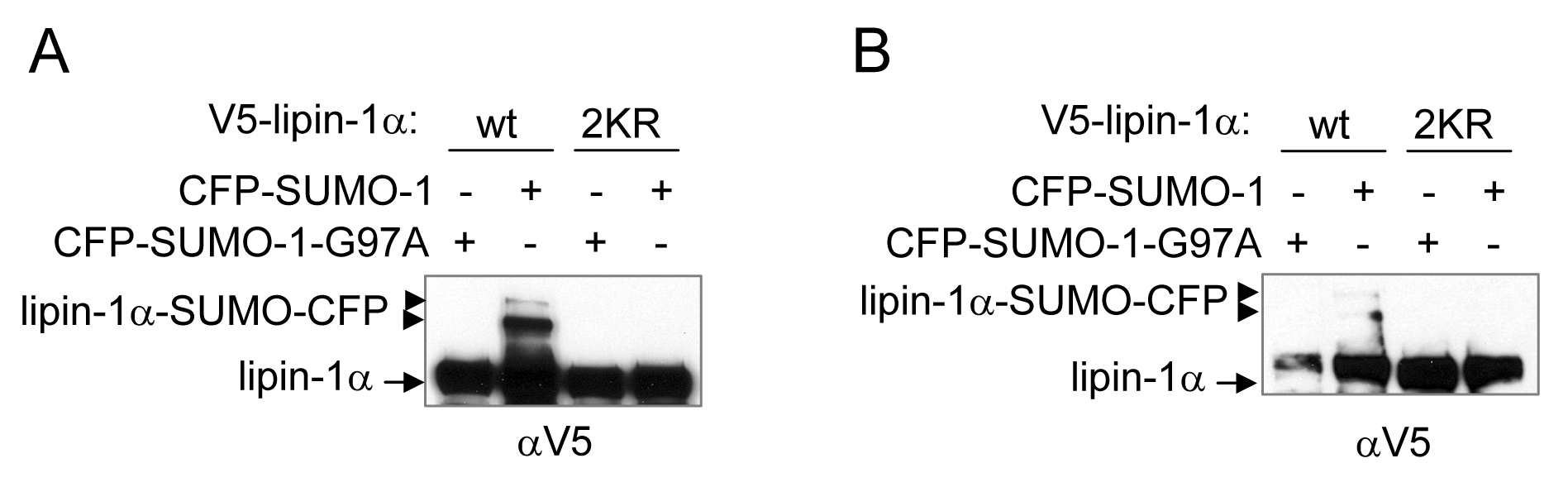

Supplement: Figure S2 — Sumoylation of lipin-1α and lipin-1α-2KR in neuronal cells. SH-SY5Y cells (A) or cortical neurons (B) were cotransfected with V5-lipin-1α or V5-lipin-1α-2KR, together with CFP-SUMO-1 or CFP-SUMO-1-G97A as indicated. 48 h after transfection, the cells were directly lysed in Laemmli sample buffer supplemented with 10 mM NEM and analyzed by immunoblotting with anti-V5 antibody. The arrowheads indicate the slower migrating form of lipin-1α. (0.12 MB TIF) [file pone.0007031.s002.tif]

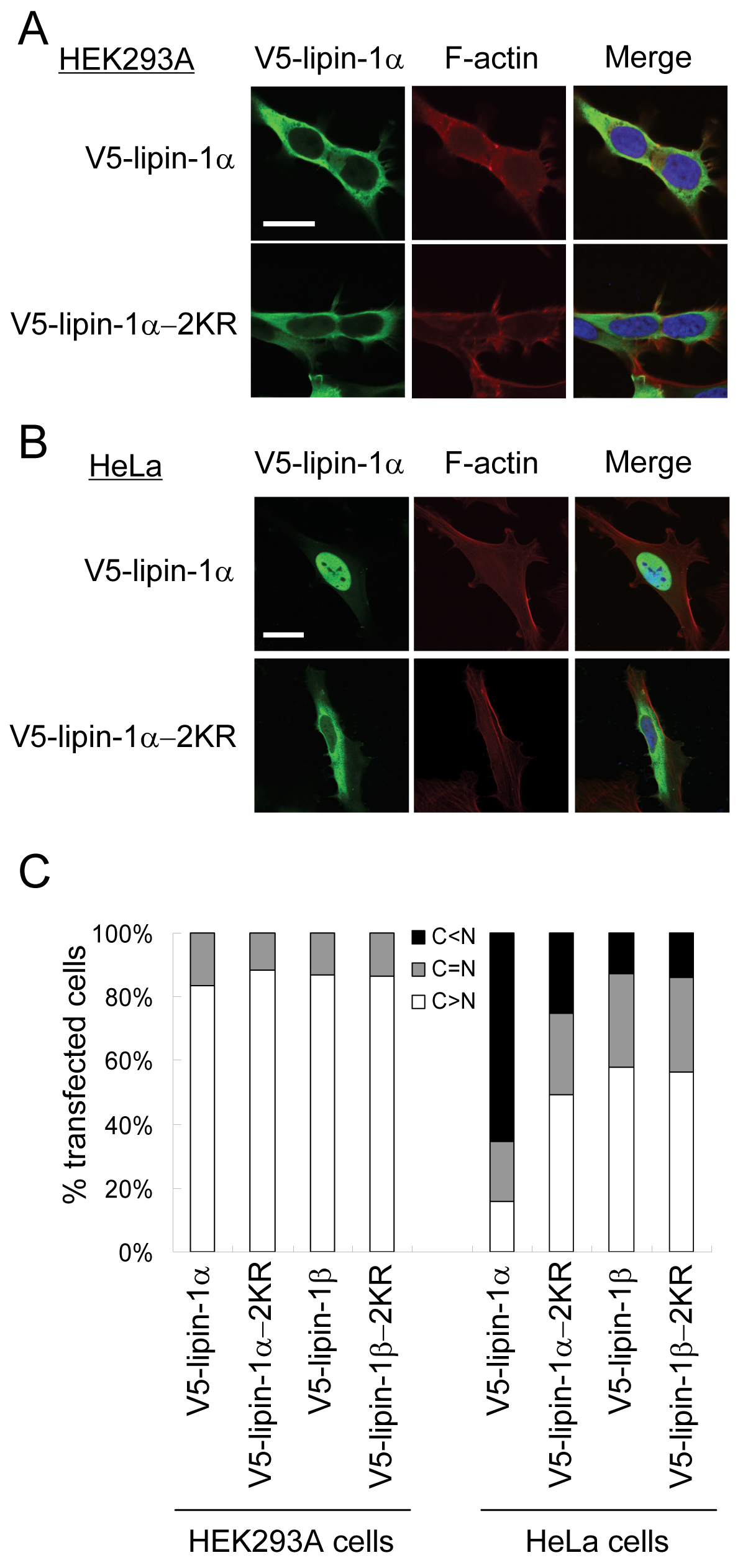

Supplement: Figure S3 — Localization of lipin-1α and lipin-1α-2KR in HEK293A and HeLa cells. HEK293A cells (A) or HeLa cells (B) were transfected with lipin-1α and lipin-1α-2KR. 48 h later, the cells were subjected to immunostaining with anti-V5 antibody (green). F-actin (red) and nucleus (blue) were stained with Alex598-Phalloidin and Hoechst 33342, respectively. Bar, 10 µm. (C) The distribution patterns of V5-lipin-1α, V5-lipin-1β, or their mutants were scored for almost 100 cells and classified into three categories: C>N, cytoplasmic-dominant distribution; N = C, roughly equal distribution in nuclear and cytoplasmic compartments; and C<N, nuclear-dominant distribution. (1.03 MB TIF) [file pone.0007031.s003.tif]

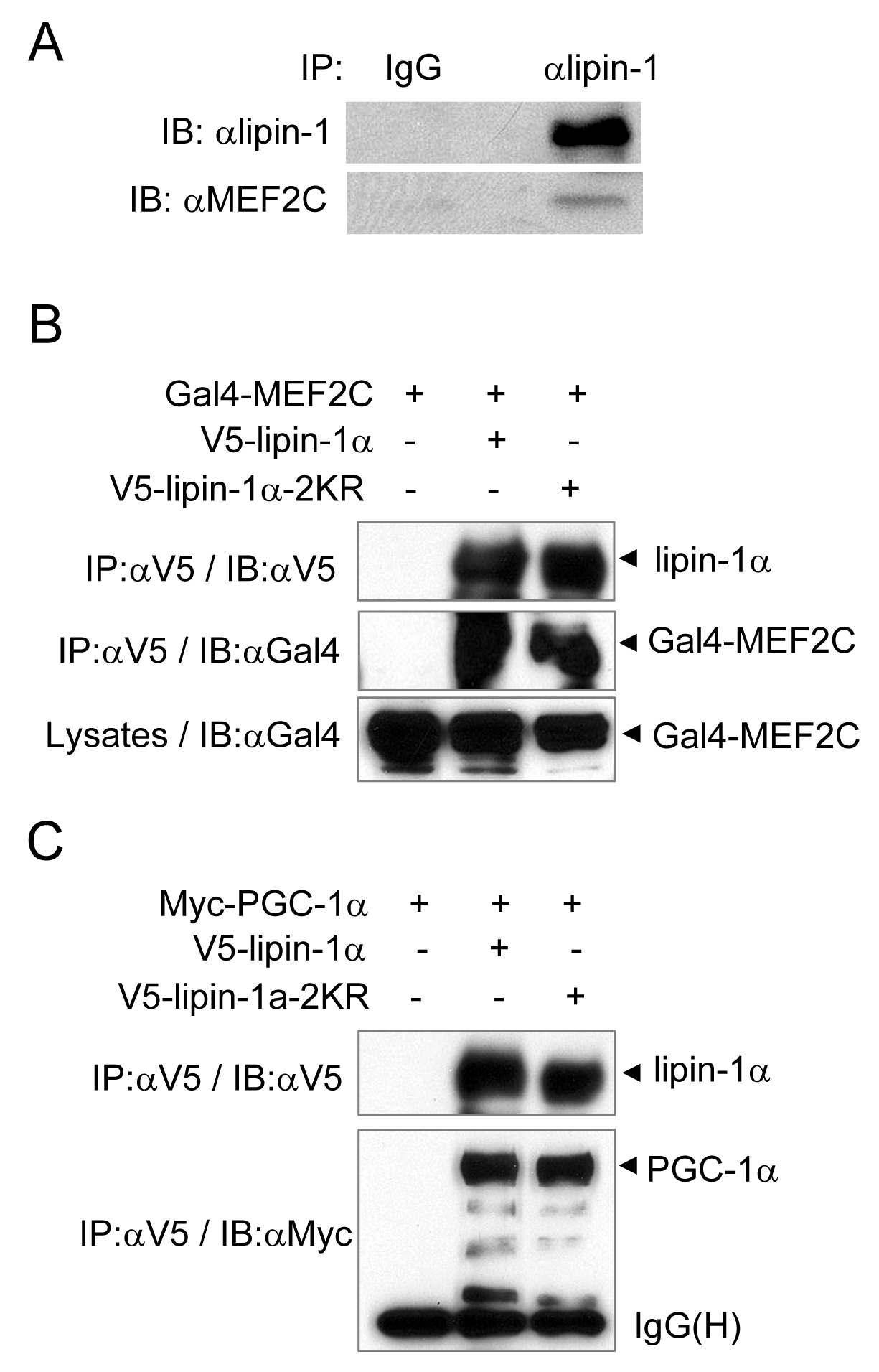

Supplement: Figure S4 — Sumoylation site lipin-1α mutant retains the capacity to associate with MEF2C and PGC-1α. (A) Mouse brain extract was prepared and immunoprecipitated with anti-lipin-1 antibody or a control IgG, and the MEF2C present in the immunoprecipitates was analyzed by immnoblotting. (B, C) HEK293A cells were transfected with V5-lipin-1α or V5-lipin-1α-2KR, together with Gal4-MEF2C (B) or Myc-PGC-1α (C). 48 h later, the cell lysates were subjected to immunoprecipitation with anti-V5 agarose, and the presence of Gal4-MEF2C (B) or Myc-PGC-1α (C) in the immunoprecipitates was analyzed by immunoblotting with anti-Gal4 or anti-Myc antibody. (0.33 MB TIF) [file pone.0007031.s004.tif]

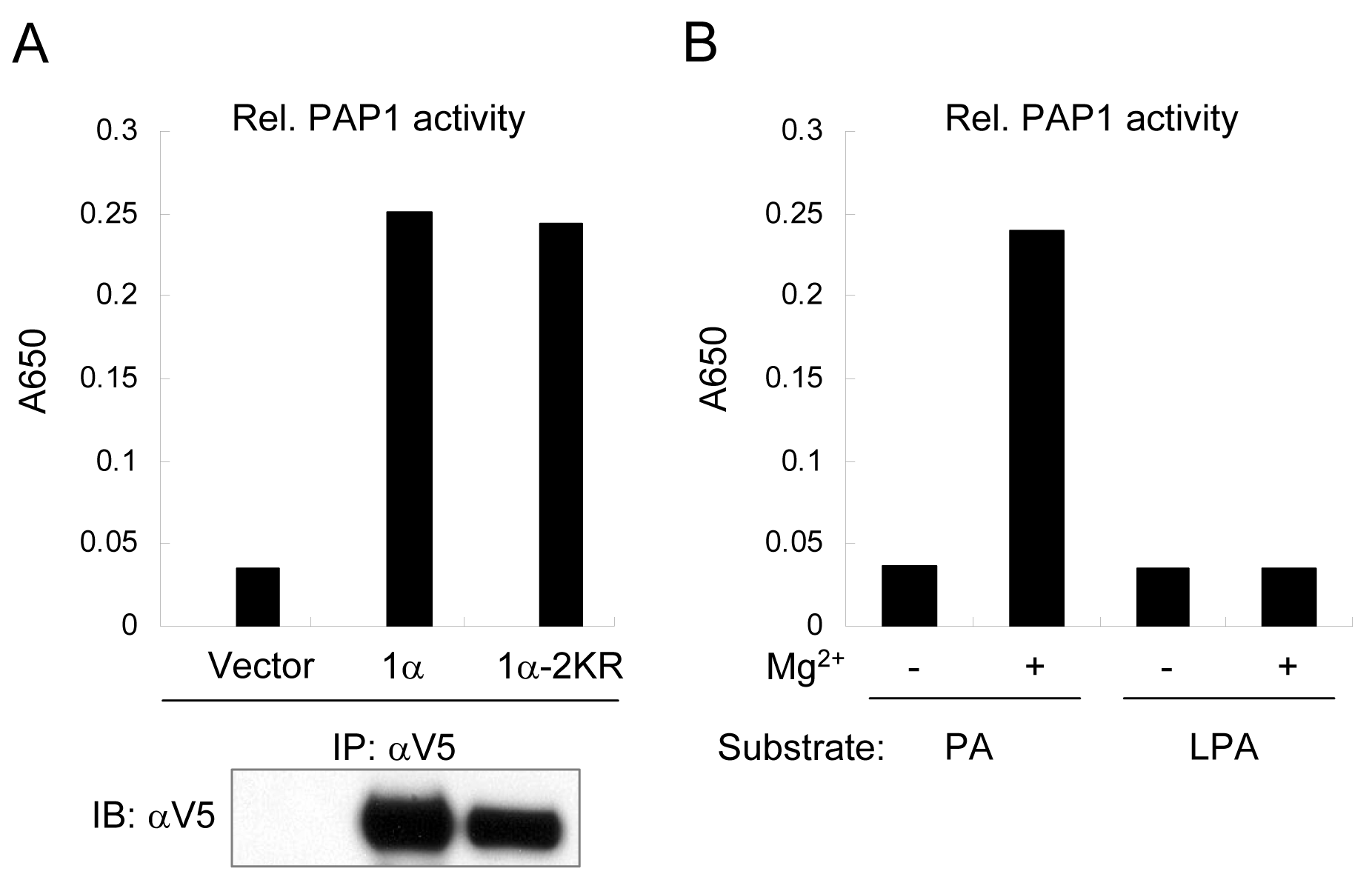

Supplement: Figure S5 — Lipin-1α and lipin-1α-2KR have comparable PAP1 activity in stably transfected SH-SY5Y cells. (A) V5-tagged lipin-1α or lipin-1α-2KR was immunopurified from SH-SY5Y cells and then subjected to the PAP1 activity analysis using PA as the substrate. Bottom: The expression of immunopurified lipin-1α or lipin-1α-2KR was determined by immunoblotting with anti-V5 antibody. (B) Immunopurified lipin-1α was subjected to the PAP1 activity analysis using PA or LPA as the substrate, in the presence or absence of Mg2+. (0.12 MB TIF) [file pone.0007031.s005.tif]
